# Highly Accurate and Fully Automatic 3D Head Pose Estimation and Eye Gaze Estimation Using RGB-D Sensors and 3D Morphable Models

**DOI:** 10.3390/s18124280

**Published:** 2018-12-05

**Authors:** Reza Shoja Ghiass, Ognjen Arandjelovć, Denis Laurendeau

**Affiliations:** 1 Computer Vision and Systems Laboratory, Laval University, 1665 Rue de l’Universite, Universite Laval, Quebec City, QC G1V 0A6, Canada; reza.shoja@gmail.com; 2 School of Computer Science, University of St Andrews, St Andrews, KY16 9SX Scotland, UK; ognjen.arandjelovic@gmail.com

**Keywords:** 3D morphable models, 3D head pose estimation, 3D eye gaze estimation, iterative closest point, RGB-D sensors

## Abstract

This work addresses the problem of automatic head pose estimation and its application in 3D gaze estimation using low quality RGB-D sensors without any subject cooperation or manual intervention. The previous works on 3D head pose estimation using RGB-D sensors require either an offline step for supervised learning or 3D head model construction, which may require manual intervention or subject cooperation for complete head model reconstruction. In this paper, we propose a 3D pose estimator based on low quality depth data, which is not limited by any of the aforementioned steps. Instead, the proposed technique relies on modeling the subject’s face in 3D rather than the complete head, which, in turn, relaxes all of the constraints in the previous works. The proposed method is robust, highly accurate and fully automatic. Moreover, it does not need any offline step. Unlike some of the previous works, the method only uses depth data for pose estimation. The experimental results on the Biwi head pose database confirm the efficiency of our algorithm in handling large pose variations and partial occlusion. We also evaluated the performance of our algorithm on IDIAP database for 3D head pose and eye gaze estimation.

## 1. Introduction

Head pose estimation is a key step in understanding human behavior and can have different interpretations depending on the context. From the computer vision point of view, head pose estimation is the task of inferring the direction of head from digital images or range data compared to the imaging sensor coordinate system. In the literature, the head is assumed to be a rigid object with three degrees of freedom, i.e., the head pose estimation is expressed in terms of yaw, roll and pitch. Generally, the previous works on head pose estimation can be divided into two categories: (i) the methods based on 2D images; and (ii) depth data [1]. The pose estimators based on 2D images generally require some pre-processing steps to translate the pixel-based representation of the head into some direction cues. Several challenges such as camera distortion, projective geometry, lighting or changes in facial expression exist in 2D image-based head pose estimators. A comprehensive study of pose estimation is given in [1] and the reader can refer to this reference for more details on the literature.

Unlike the 2D pose estimators, the systems based on 3D range data or their combination with 2D images have demonstrated very good performance in the literature [2,3,4,5,6,7]. While most of the work on 3D pose estimation in the literature is based on non-consumer level sensors [8,9,10], recent advances in production of consumer level RGB-D sensors such as the Microsoft Kinect or the Asus Xtion has facilitated the design and implementation of real-time facial performance capture systems such as consumer-level 3D pose estimators, 3D face tracking systems, 3D facial expression capture systems and 3D eye gaze estimators. In this paper, we focus on the recent 3D pose estimators and tracking systems and their application in appearance-based eye gaze estimation systems using consumer level RGB-D sensors.

### 1.1. Related Work on 3D Pose Estimation Using RGB-D Sensors

The 3D head pose estimation systems can be divided into three categories: (i) statistical approaches; (ii) model-based posed estimation methods; and (iii) facial feature-based pose estimation techniques [11]. Each of these approaches comes with their specific limits and advantages. Statistical methods may need a large database for training a regressor. However, they can estimate the subject head pose on air, i.e., the system can estimate the head pose for each frame even in a shuffled video sequence. In contrast, model-based approaches generally need an offline step for subject-specific head model reconstruction with significant subject cooperation. Next, a point cloud registration technique such as rigid/non-rigid ICP should be used to register the model with depth data. In other words, unlike the supervised learning based approaches, they are generally based on tracking. Thus, re-initialization becomes a challenge. Facial feature-based pose estimation techniques try to track facial features or patches, which, in turn, can help in calculation of pose using techniques such as PnP [10] or encoding the face 3D shape using view-invariant descriptors and infer head pose through matching [12].

To the best of our knowledge, one of the most important works on pose estimation using consumer level *RGB-D sensors* is the work of Fanelli et al. [2,3]. As the authors provided a ground truth data and a database for comparison, their work has become the gold standard for comparison in the literature. Their work falls in the category of statistical approaches. In their work, the authors proposed a pose estimation system based on Random Forests. For the evaluation of their system, they acquired a database of 20 subjects, which is called the Biwi head pose database. Next, they divided the database into a training and test sets. Afterwards, a commercial face tracker is used for annotation of the training set, i.e., a subject-specific head model is constructed using the commercial system to match each person’s identity and track the head in training depth frames. The commercial tracker measures a subject’s 3D head locations and orientations, which, in turn, are used to train their regression based system. Finally, some patches of fixed size from the region of the image containing the head as positives samples, and from outside the head region as negatives were randomly selected for training the system. A major limitation of this system is that it requires an offline training phase with subject cooperation. Moreover, the performance of the system in the testing phase is subject to the output of the commercial head tracker in the training phase. In [3], the authors continued their previous work [2] by creating a dataset of synthetic depth images of heads, and extracting the positive patches from the synthetic data, while using the original depth data to to extract negative patches. A drawback of this system is the limited number of synthetic models and negative patches for performing a regression task, without learning subject’s own head [3]. Ref. [13] proposed a system based on cascaded tree classifiers with higher accuracies than Fanelli et al. [8] proposes a 3D face tracker based on particle filters. The main idea in their system is the combination of depth and 2D image data in the observation model of the particle filter.

### 1.2. Related Work on 3D Gaze Estimation Using RGB-D Sensors

**Figure 1:**
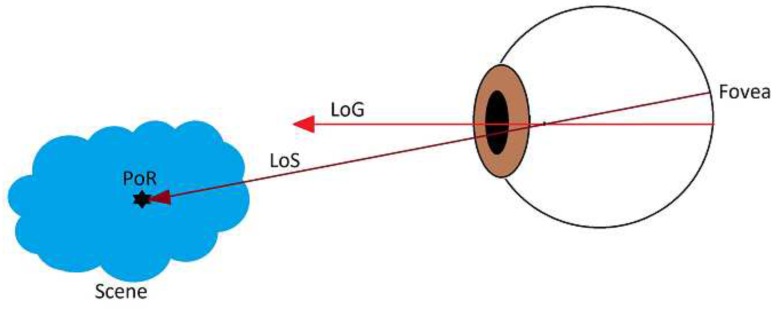
A simplified Schematic of Human Eye with LoS, GoS and PoR.

Based on the context, the term *Gaze Estimation* can be interpreted as one of the following closely related concepts: (i) 3D Line of Sight (LoS); (ii) 3D Line of Gaze (LoG); and (iii) Point of Regard (PoR). Within the eyeball coordinate system, the LoG is simply the optical axis, while the LoS is the ray pointing out from fovea and eyeball rotation center. The PoR is a 3D point in the scene to which the LoS points. Figure 1 demonstrates a simplified schematic of human eye with LoS, GoS and PoR. In the literature non-intrusive gaze estimation approaches generally fall into one of the following categories: (i) feature-based approaches; and (ii) appearance-based approaches [14].

Feature-based approaches extract some eye specific features such as eye corner, eye contour, limbus, iris, pupil, etc. These features may be aggregated with the reflection of external light setup on the eye (called glints or Purkinje images) to infer the gaze. These methods are generally divided into two categories: (i) model-based (geometric); and (ii) interpolation-based.

Model based methods rely on the geometry of the eye. These methods directly calculate the point of regard by calculating the gaze direction (LoS) first. Next, the intersection of the gaze direction and the nearest object of the scene (e.g., a monitor in many applications) generates the point of regard. Most of the model-based approaches require some prior knowledge such as the camera calibration or the global geometric model of the external lighting setup [15,16,17,18,19,20].

Unlike the model-based methods, the interpolation-based approaches do not perform an explicit calculation about the LoS. Instead, they rely on a training session based on interpolation (i.e., a regression problem in a supervised learning context). In these methods, the feature vector between pupil center and corneal glint is mapped to the corresponding gaze coordinates on a frontal screen. The interpolation problem is formalized using a parametric mapping function such as a polynomial transformation function. The function is used later to estimate the PoR on the screen during the testing session. A calibration board maybe used during the training session [21,22,23,24,25,26,27]. The main challenge with the interpolation-based approaches is that they can not handle the head pose movements [14]. Notice that feature-based approaches in general need high resolution images to precisely extract the eye specific features as well as the glints. Moreover, they may require external lighting setups which are not ubiquitous. This motivates the researchers to train appearance-based gaze estimators, which rely on low quality eye images (a holistic-based approach instead of feature-based). However, appearance-based approaches generally have less accuracy.

As opposed to feature-based approaches, appearance-based methods do not rely on eye-specific features. Instead, they learn a one-to-one mapping from the eye appearance (i.e., the entire eye image) to the gaze vector. Appearance-based methods do not require camera calibration or any prior knowledge on the geometry data. The reason is that the mapping is made directly on the eye image, which makes these methods suitable for gaze estimation from low resolution images, but with less accuracy. In this context, they share some similarities with interpolation-based approaches. Similar to the the interpolation-based methods, appearance-based methods do not handle the head pose.

Baluja and Pomerleau [28] first used the interpolation concept from image content to the screen coordinate. Their method is based on training a neural network. However, their method requires more than 2000 training samples. To reduce such a large number of training examples, Tan et al. [29] proposed using a linear interpolation and reconstructed a test sample from the local appearance manifold within the training data. By exploiting this topological information of eye appearance, the authors reduced the training samples to 252. Later, Lu et al. [30] proposed a similar approach to exploit topological information encoded in the two-dimensional space of gaze space. To further reduce the number of the training data, Williams et al. [31] proposed a semi-supervised sparse Gaussian process regression method S3GP. Note that most of these methods assumed a fixed head pose. Alternatively, some other researchers used head mounted setups, but these methods are no longer non-intrusive [32,33].

With the main intention of designing a gaze estimator robust to head pose, Funes and Odobez [7,34] proposed the first model-based pose estimator by building a subject-specific model-based face tracker using Iterative Closest Point (ICP) and 3D Morphable Models. Their system is not only able to estimate the pose, but is also able to track the face and stabilize it. A major limitation of their method is the offline step for subject specific 3D head model reconstruction. For this purpose, they manually placed landmarks (eye corners, eyebrows, mouth corners) on RGB image of the subject, and consequently added an extra term to the cost function in their ICP formulation. In other words, their ICP formulation is supported by a manual term. Moreover, the user has to cooperate with the system and turn their head from left to right. Recently, the authors proposed a more recent version of their system in the work of [11] without the need for manual intervention.

### 1.3. Contribution of the Proposed Approach

Unlike [2,3,7,34], our proposed system does not require any commercial system to learn a subject’s head or any offline step. A key contribution of our approach is to propose a method to automatically learn a subject’s 3D face rather than the entire 3D head. Consequently, we no longer need subject’s cooperation (i.e., turning their head from left to right), which is important in previous works for model-based pose estimation systems. In addition, unlike [7], our system does not require any manual intervention for model reconstruction. Instead, we rely on Haar features and boosting for facial feature detection, which, in turn, can be used for face model construction. Note that we use only one RGB frame for model reconstruction. The tracking step is based on depth frames only. After learning a subject’s face, the pose estimation task is performed by a fully automatic, user non-cooperative and generic ICP formulation without any manual term. Our ICP formulation is robustified with Tukey functions in tracking mode. Thanks to the Tukey functions, our method successfully tracks a subject face in challenging scenarios. The outline of the paper is as follows: The method details are explained in Section 2. Afterwards, the experimental results are discussed in Section 3. Finally, the conclusions are drawn in Section 4.

## 2. Method Details

**Figure 2:**
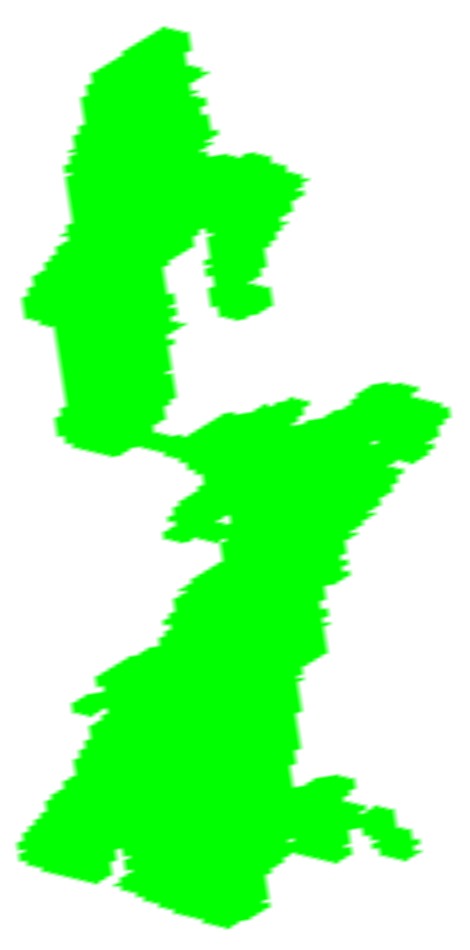
Depth data obtained from the first frame and visualized from profile. They consist of both the facial part and data from other body parts.

Our method consists of four key steps: (i) geometry processing of a generic face model and the first depth frame; (ii) generic face model initialization (i.e., model positioning at the location of the head); (iii) subject-specific face model construction by morphing the initialized generic model; and (iv) tracking the face in the next depth frames using the subject-specific face model. In our proposed system, we only model the face of the subject rather than the entire head, which, in turn, helps us to design a very robust, accurate and non-cooperative head tracking and pose estimation system. To accomplish this goal, a generic model is positioned on the subject’s face in depth data. Next, it learns the subject’s face and finally starts to track it. Both *positioning* a generic model on the subject’s face and *tracking* it in depth data are accomplished using an ICP-based technique. However, the ICP registration technique which serves for positioning faces a major challenge: the generic model is a model of a complete head and not only the face. On the other hand, the depth data contain not only the face of the subject, but also other body parts such as the torso or the background (Figure 2). Note that a major difficulty with ICP is its sensitivity to outliers and missing data between two 3D point clouds. To tackle this problem in model initialization, we perform geometry processing, which is explained next.

### 2.1. Geometry Processing

**Figure 3:**
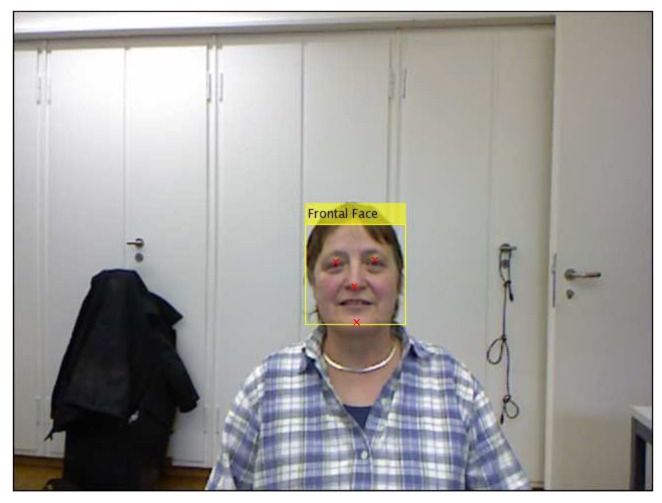
Face and facial features detection from the first RGB frame of a subject.

In this step, the goal is trimming the depth data and the generic model to remove spurious data and outliers. Note that we perform this step on the first depth frame only. The reason is that we should initialize (position) the model at the position of the subject’s head, before tracking starts. For this purpose, we capture the entire environment using the Kinect. Next, we filter out the spurious point cloud and just keep the region of interest in the first depth frame, i.e., the facial surface. For this purpose, the first depth frame is automatically trimmed to discard the residual data. To this end, we need to automatically detect and localize the facial features (i.e., eyes, nose, and mouth) on the point cloud to determine the way the depth data should be trimmed. Detection of the facial features from a noisy depth frame directly is a challenge. Fortunately, the Kinect provide us the first RGB frame. Thus, the face and facial features are detected on the first RGB frame by first using Haar features and boosting [35]. Figure 3 demonstrates an example of the face and facial feature detection. As some false detections may occur, the next step is to reject them automatically. This is accomplished by utilizing the prior knowledge about the structure of a face and the relative positions of eyes, nose and mouth on a detected face.

**Figure 4:**
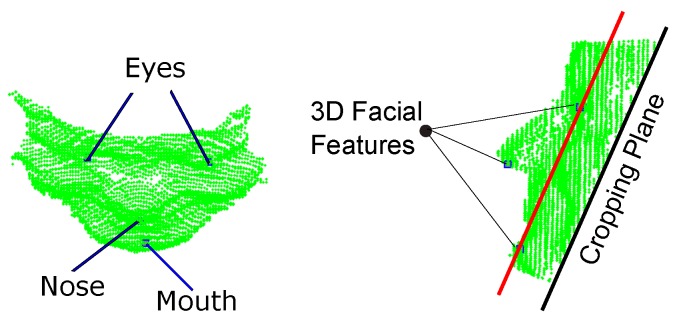
Trimming the first depth frame: (**left**) top view; and (**right**) profile view.

After the features are detected on the first RGB frame, their 3D loci are determined on the first depth frame through back projection using Kinect calibration data. To trim the depth data, a 3D plane passing through the 3D coordinates of the eyes and mouth is defined and shifted by an offset equal to the distance between the left and right eye. The shifted plane is called *the cropping plane*. Next, the depth data beneath the plane are discarded. Figure 4 shows the 3D loci of the facial features on the corresponding depth data trimmed by the cropping plane.

**Figure 5:**
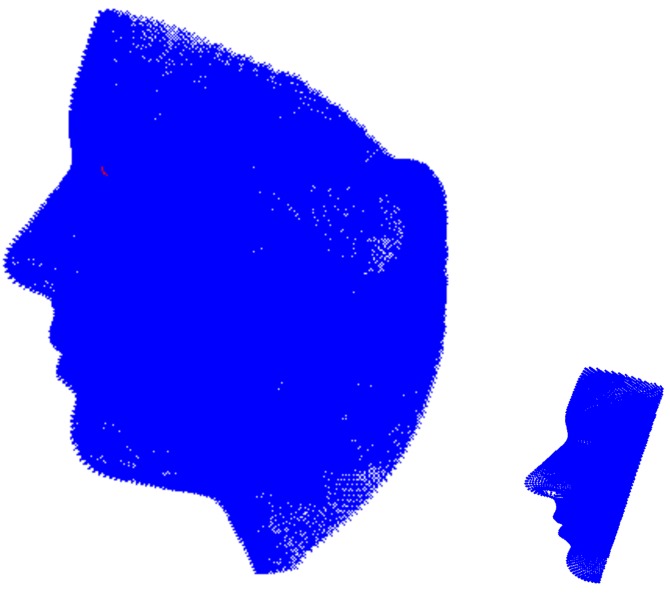
Trimming the 3D Morphable model mean shape: (**left**) before trimming; and (**right**) after trimming.

Once the subject’s face is captured and trimmed in 3D, the next step is to construct a model which simulates the subjects face (rather than the complete head). The type of 3D model we use to simulate the subject’s identity is a family of Active Appearance Models (AAMs) called 3D Morphable models. Using these models, a subject’s 3D head scan can be reconstructed by adding a set of weighted principal components (PCs) to the mean shape (the mean shape is the mean of all of the 200 subject’s face scans in the database). For instance, we focus on the mean shape of the model. Similar to trimming the depth data, the mean shape of the 3D Morphable model is trimmed to facilitate the procedure of subject specific model construction through registration. In this context, a plane similar to that in Figure 4 is fitted to the model’s mean shape. Once the mean shape is trimmed, it should be scaled to the size of the subject’s face in 3D space. To this end, the model is scaled so the distance between the left and right eyes of the model and that of the subject’s face scan (i.e., the first depth frame) becomes equal. Figure 5 demonstrates the mean shape of model, **m**, before and after trimming.

### 2.2. Generic Model Positioning

**Figure 6:**
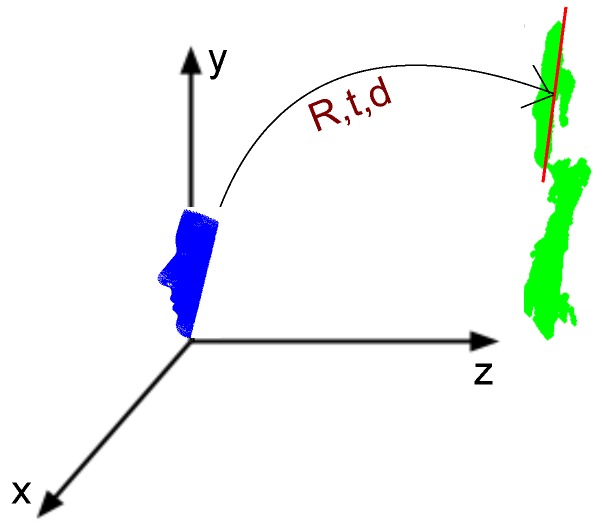
Positioning the model on the face. The blue object is the trimmed model, while the green object is the subject scanned by the Kinect.

After processing both model and depth data, the trimmed model is positioned on the face of the subject using rigid ICP. Figure 6 demonstrates this step for the model and first depth frame. After initializing the generic model on the subject’s face, the model is ready to morph and learn the subject’s face (Section 2.3) and track it afterwards (Section 2.4).

### 2.3. Learning and Modeling the Subject’s Face

Capturing the subject’s facial shape variations via morphing the mean shape is the main objective of this step. This problem can be considered as finding the weights of shape PCs in the 3D Morphable model, where each weight describes the contribution of its corresponding PC in simulating a subject’s face. Similar to the generic ICP problem, this part also can be described by minimization of a cost function. Thus, we can unify both ICP and PCA terms into a unique equation and reformulate a more generic ICP problem through minimizing the following energy function [36]:

(1) $$\begin{array}{cc} E\left(Z,d,R,t\right) & =\omega_{1}E_{match}+\omega_{2}E_{rigid}++\omega_{3}E_{model}\\ & E_{match}=\sum_{i=1}^{n}{\left(N_{i}^{T}\left(z_{i}-C_{Y}\left(z_{i}\right)\right)\right)}^{2}\\ & E_{rigid}=\sum_{i=1}^{n}∥z_{i}-\left(Rx_{i}+t\right){∥}_{2}^{2}\\ & E_{model}=\sum_{i=1}^{n}∥z_{i}-\left(P_{i}d+m_{i}\right){∥}_{2}^{2} \end{array}$$

where *Y* is the target surface in $R^{3}$, *X* is the source surface, and *Z* is a deformed version of *X* which should be aligned with *Y*. Notice also that $C_{y}\left(z_{i}\right)$ is the closest point in the target surface to the point $z_{i}$ (i=1,2,…,n, where *n* is the number of points in source). In this equation, the first term is the point-to-plane matching error, the second term is the point-to-point matching error, while the third term is the model error (for more details about these error the reader is referred to [36]). The energy function can be minimized by linearizing Equation (1) and iteratively solving the following linear system:

(2) $$\begin{array}{c} \underset{Z_{i}^{t+1},d,\tilde{R},\tilde{t}}{\mathrm{argmin}}\;\sum_{i=1}^{n}\omega_{1}{\left(n_{i}^{T}\left(z_{i}^{t+1}-C_{Y}{\left(z_{i}\right)}^{t}\right)\right)}^{2}+\\ \omega_{2}∥z_{i}^{t+1}-\left(\tilde{R}\left(Rx_{i}+t\right)+\tilde{t}\right){∥}_{2}^{2}+\\ \omega_{3}∥z_{i}^{t+1}-\left(P_{i}d+m_{i}\right){∥}_{2}^{2} \end{array}$$

where *t* is the number of iterations, $z_{i}^{0}=x_{i}$, *d* contains the weights of PCs, and $\tilde{R}$ and $\tilde{t}$ are the linear updates that we obtain for the rotation (**R**) and translation (**t**) matrices, respectively, at each iteration. Notice that $n_{i}$ is the normal to the surface at point $C_{Y}{\left(z_{i}\right)}^{t}$, i.e., point to plane matching error. For more details, the reader is referred to the tutorial by Bouaziz and Pauly [36].

### 2.4. 3D Head Tracking and Pose Estimation

Once the model is constructed from the first depth frame, the pose (orientation alone) of the head can be calculated directly from the rotation matrix, *R*, in terms of roll, pitch and yaw [37]. In Section 2.3, the rotation matrix corresponding to the first depth frame of the subject is obtained during model construction. A question arises here: How can one obtain the rotation matrices for the next depth frames? Indeed, this question is addressed by 3D registration of the form of Equation (1) with some differences. The first difference is that we no longer need to capture the subject’s face variations, *d*, because it is calculated only once for the entire procedure. Thus, the $E_{model}$ term is dropped from Equation (1). The other difference is that we no longer need to trim the next depth frames. The reason is that the model is already fitted to the first depth frame during model construction (see Figure 6) and we expect the system to work in tracking mode. In tracking mode, head displacement in the next frame compared to the current frame is small and the model displacement should be very small compared to the initialization mode. Thus, instead of trimming the next depth frames, one can take advantage of registration using *Tukey functions*, which will filter out bad correspondences with large distances. The pose estimation procedure for the next depth frames is as follows: for the second depth frame, the model rotation and translation increments are calculated relative to that of the first depth frame. Next, the rotation and translation matrices for the second depth frame are obtained by applying the updates to the rotation and translation matrices in the first depth frame. This procedure is continued for the next frames. For each frame, the head pose can be directly calculated from the rotation matrix in terms of pitch, yaw and roll.

Robustness of Registration to Outliers

As mentioned, partial overlaps among source, target and outliers in the data are the most challenging problems in registration through ICP [38]. Two types of outliers exist: (i) outliers in the source point cloud; and (ii) outliers in the target point cloud. Discarding unreliable correspondences between the source and the target is the most common way to handle this problem. In Section 2.2, this goal was accomplished by trimming both model and depth data in the first depth frame. However, for the 3D face tracking mode, the same method cannot be used because the initialization modality is based on detection of facial features. Applying facial feature detection for each frame can decrease the frame rate at which the system operates. On the other hand, a limit of our method is that the system cannot start from an extreme pose, as the facial feature detection algorithms will fail. Fortunately, as the model is already positioned onto the face of the subject, we no longer need to trim the upcoming depth frames to perform tracking using ICP. Instead, we use Tukey functions to robustify the ICP. Tukey functions assign less weight to the bad correspondences and decrease or remove their effect on the energy function.

Robustness of Registration to Extreme Pose

A question may arise at this point: Can the method handle the case of a face with extreme pose where most facial parts cannot be sensed by the Kinect? In this case, the model should be registered with a partial point cloud of the subject’s face. This leads to increasing the number of points in the source (trimmed model) without good correspondences in the target (partial point cloud of face). As a result, such points will form bad correspondences with relatively large Euclidean distance values. Fortunately, we also address this problem by using robust functions in the tracking mode, as robust functions discard/decrease the effect of such bad correspondences in the energy function. To clarify this, notice that bad correspondences inherently produce large Euclidean distances, while this is not the case for good correspondences. On other other hand, narrow robust functions act as low pass filters and discard the bad correspondences.

Robustness of Registration to Facial Expression Changes

As we use rigid ICP, facial expression changes may be considered as a challenging factor. In this context, Funes and Odobez [7] used a mask and only considered the upper part of the face in the rigid registration part of their system. Notice that we do not use such a mask. The reason is that, most of the time, the subject may not show *significant* facial expression changes (such as laughing or opening the mouth). On the other hand, relying on more data of the face may result in a more robust registration task. The problem becomes more challenging if we consider that self-occlusion may occur on the upper part of the face. For these reasons, we prefer not to use a mask. Instead, we rely on the robust Tukey functions to improve the robustness in the case of facial expression changes.

#### 2.4.1. Head Pose Stabilization

**Figure 7:**
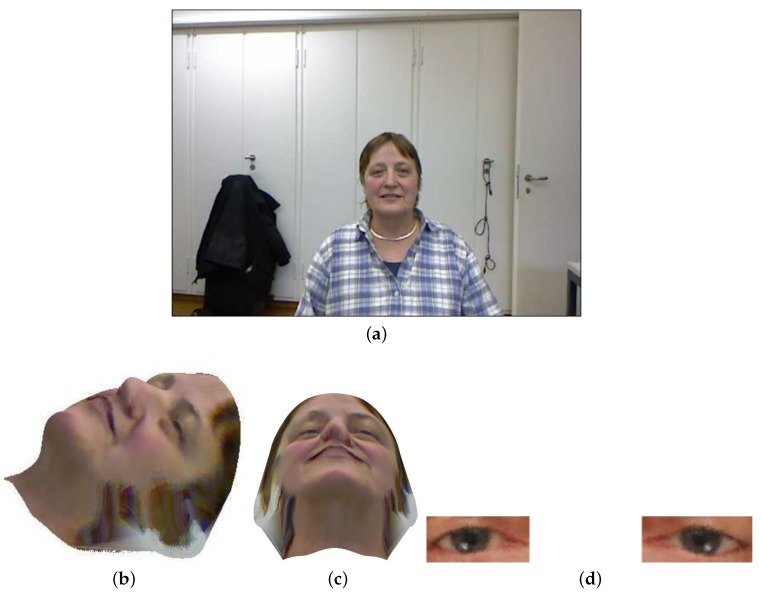
Applying the face texture from: (**a**) an RGB frame to the subject’s specific model. (**b**) The model visualized from side view; and (**c**) the same model visualized from down view. The left and eye appearances (**d**) are some examples of pose-free eye appearance which can be used to train an interpolation function.

This step is a pre-processing step for gaze estimation. First, we assume that the extrinsic parameters from the camera coordinates to the 3D depth sensor coordinates are known. As soon as the head pose is calculated, the texture of the corresponding RGB frame can be back-projected to a pose free 3D head model. This pose free head model can be visualized from any direction (e.g., see Figure 7b,c), but the ideal direction is the frontal view. The next step is to crop the eye appearances from this frontal view (e.g., see Figure 7d).

### 2.5. Gaze Estimation: Point of Regard

In this paper, gaze estimation refers to estimation of the point of regard (PoR). Our gaze estimation system consists of two parts: (i) training; and (ii) testing. The goal of the training part is to learn the parameters of an interpolation function which maps the pose-free eye appearance to the gaze vectors. In the training phase, the gaze vectors are known, because a computer screen is used to serve as a calibration pattern. At each time step, a moving target is displayed on the screen, where the subject’s eye locations are known thanks to the model-based head tracker (Section 2.4).

On the other hand, the goal of the testing phase is estimating the PoR for a new eye–appearance. As mentioned previously, the PoR is the intersection of the line of sight with an object in the world coordinate system (WCS). The gaze estimation problem can be divided into four parts: (i) head pose estimation in the world coordinate system; (ii) line of sight estimation in the head coordinate system; (iii) line of sight calculation in the world coordinate system; and (iv) intersecting the line of sight with the object (i.e., a display in our case) in the WCS.

#### 2.5.1. Training

**Figure 8:**
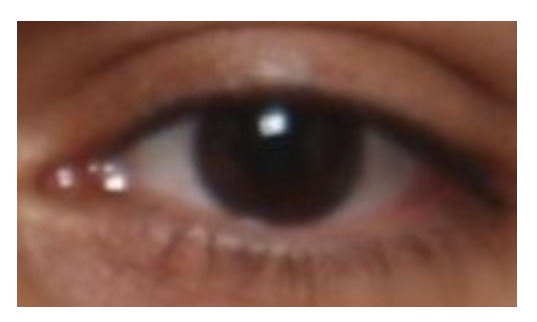
An exemplar pose-free eye appearance which looks forward.

In this section, we want to train an appearance-based gaze estimator to calculate a one to one mapping between the pose-free eye appearance (i.e., eye image) and its corresponding gaze vector in the head coordinate system. As an example, Figure 8 demonstrates a pose-free eye appearance which looks directly to the front (i.e., a 0 degree gaze vector).

Let us assume that $\left[\left(I_{1},G_{1}\right),\left(I_{2},G_{2}\right),\ldots ,\left(I_{N},G_{N}\right)\right]$ are the training data, where $I_{i}$ and $G_{i}$ (i=1,2,…,N) stand for the eye appearance image and their corresponding gaze vectors, respectively. It is possible to train an interpolation-based gaze estimator using these *N* training examples. For any new $G_{t}est$, it is possible to estimate the gaze by interpolation. Note that, in appearance-based gaze estimators, the $G_{i}$ (i=1,2,…,N) vectors are calculated using a calibration pattern [7,30,34]. In this paper, the same methods explained in [7,30,34] are for interpolation: (i) A K-NN based approach; and (ii) Adaptive Linear Regression (ALR) which is based on manifold learning. The reader is referred to [7,30,34] for further details.

#### 2.5.2. Head Pose Estimation in the WCS

This step is explained in Section 2.4. As a subject-specific model is tracking the head in the depth frames, it is possible to precisely track the location of eyes in the WCS. On the other hand, in geometry, a line can be uniquely defined with a point and a vector. Thus, the eye locations given by the head tracker are can be used to calculate the line of sight passing through them. In general, two goals are accomplished in this part: (i) estimating the pose (i.e., both position and orientation) of the head and eyes; and (ii) head pose stabilization (See Section 2.4).

#### 2.5.3. LoS Calculation in the WCS

The LoS is estimated in the head coordinate system. On the other hand, the head is tracked using the head tracker. Thus, we can calculate the rotation and translation of the head coordinate system in the WCS. Consequently, we can re-express the line of sight in the WCS.

#### 2.5.4. PoR Calculation

Once the LoS is calculated in WCS, we can intersect it with the computer screen, which is a plane in the WCS (the position of the computer screen is known).

## 3. Experimental Evaluation

In this section, we report our empirical evaluation. We start by describing the datasets used in our experiments, follow this with an explanation of the evaluation protocol, and finish with a report of the results and their discussion.

### 3.1. Databases

#### 3.1.1. 3D Basel Face Model (BFM)

The 3D Basel Face Model (BFM) is a Morphable model calculated from registered 3D scans of 100 male and 100 female faces. The model geometry consists of 53,490 3D vertices connected by 160,470 triangles. The model is given by the following:

- The mean shape
- The 199 principal components (PCs) of shape obtained by applying PCA on 200 subjects facial shape in the database
- The variance of shape
- The mesh topology
- The mean texture
- The 199 principal components (PCs) of texture obtained by applying PCA on 200 subjects facial texture in the database
- The texture variance

**Figure 9:**
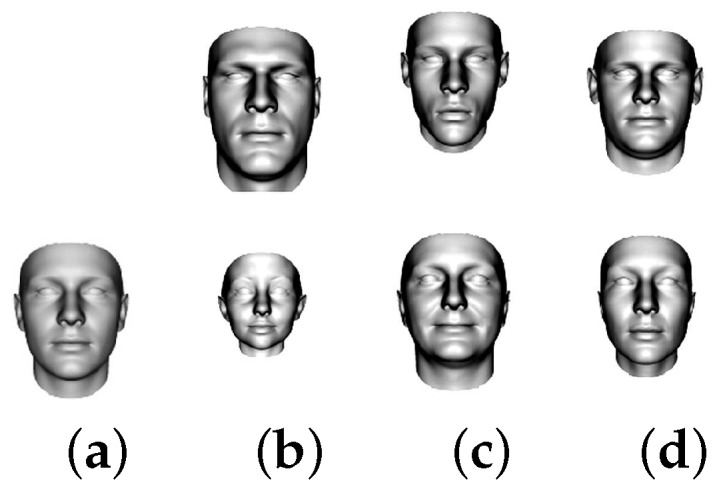
(**a**) The mean; (**b**) the first; (**c**) the second; and (**d**) the third principal components (visualized: ± 5 standard deviation) of the shape model. The images are taken from the database website

**Figure 10:**
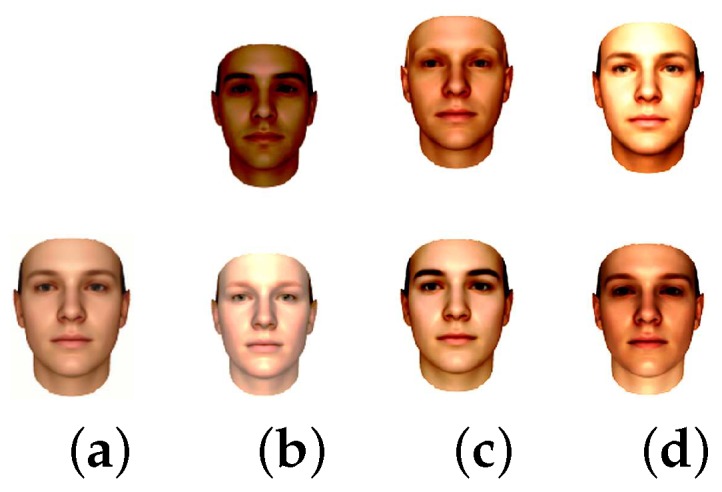
(**a**) The mean; (**b**) the first; (**c**) the second; and (**d**) the third principal components (visualized: ± 5 standard deviation) of the texture model. The images are taken from the database website.

Figure 9 and Figure 10 demonstrate the mean and the first, second and third principal components (visualized: ± 5 standard deviation) of the shape and texture model respectively.

Any unknown face can be explained as a linear combination of the principal components and the mean shape/texture. In this paper, we only use the shape dataset (i.e., shape principal components together with mean shape) for the construction of a subject’s specific face model (i.e., the head trackers).

#### 3.1.2. Biwi Kinect Head Pose Database

We used the Biwi Kinect Head Pose Database [2,3] to evaluate the effectiveness of our method for the following reasons. Firstly, to the best of our knowledge, it is the only RGB-D database for pose estimation reported in the literature. Secondly, it provides ground truth data for comparison, and we wanted to make our results directly comparable to not only those of Fanelli et al. [2,3], but also to the recent works that have used this database. The dataset contains over 15000 depth frames and RGB image of 20 people, six females and fourteen males, where four people were recorded twice. The head pose ranges through about 75 degrees yaw and 60 degrees pitch. The ground truth for head rotation is also provided by a commercial software.

#### 3.1.3. EYEDIAP Database

We used the EYEDIAP gaze database [39] to evaluate the effectiveness of *gaze estimation* part of our method for the following reasons. Firstly, to our knowledge, it is the only Kinect based database for gaze estimation in the literature. Secondly, it provides ground truth data for comparison of gaze estimation (but not pose) results. In addition, we wanted to make our results statistically comparable to the work of Funes and Odobez [7,34]. The dataset contains over 4450 depth frames and RGB image of 16 people, among them 14 subjects participated in a screen-based gaze estimation scenario. Each session itself is divided into two other sessions, where the subject was asked to keep the head stationary or moving.

### 3.2. Evaluation Methodology

#### 3.2.1. Subject Specific Model Construction

**Figure 11:**
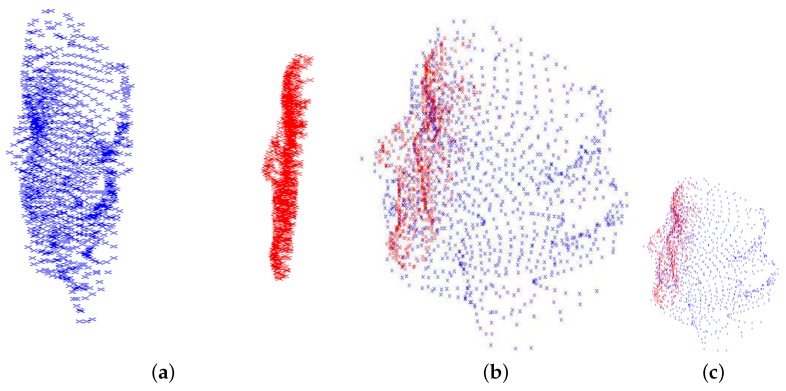
Registration procedure to capture subject’s variations, *d*, together with transformation matrices *R* and *t*: (**a**) before model initialization; (**b**) after model initialization and before capturing subject’s face variations; and (**c**) after the initialization is accomplished (ready for tracking).

We evaluated the proposed algorithm in a setting in which the first RGB frame and the first depth frame were used for learning in an unsupervised context, while the other depth frames were used for testing. Figure 11 demonstrates the registration procedure. In this figure, the blue point cloud is the (down sampled) mean shape of the BASEL data, while the red point cloud is the trimmed depth scan of the first subject in the Biwi database (the subject in Figure 3). Notice that trimming the model is not shown here, but it is considered in calculations. We wanted to register the two shapes with each other and, at the same time, capture the variation of the subject’s face by minimizing Equation (1).

#### 3.2.2. Pose Estimation and Tracking

**Figure 12:**
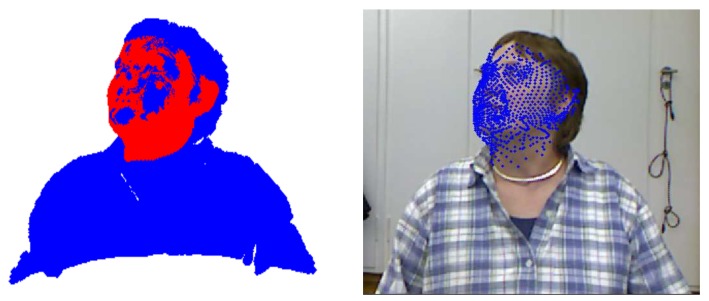
An instant of the tracking mode: the model (**red**) tracks the depth data (**blue**) and it is back-projected to the RGB frame

After the subject’s specific model was constructed from the first (trimmed) depth frame, we dropped the model term from the energy function and continued the registration of the model and depth data. The pose estimation for each frame could be directly calculated from the rotation matrix, *R*, obtained from registration. Figure 12a shows a sample where the model (red) was registered with the depth data (blue). The model was superimposed on the corresponding RGB frame through the Kinect calibration data (Figure 12b) for a better visualization.

**Figure 13:**
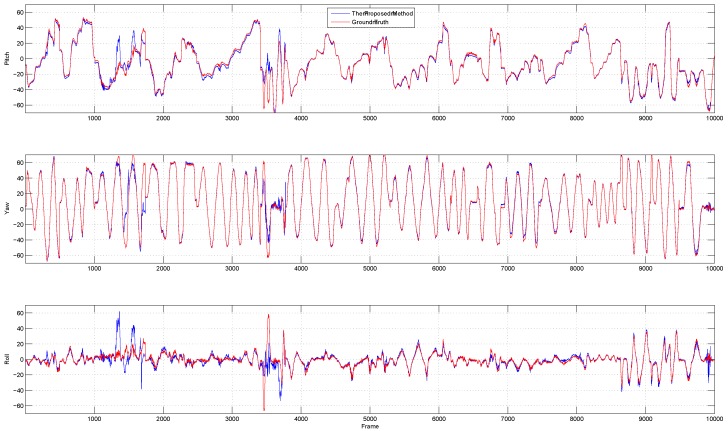
The experimental results of the proposed facial pose estimator on Biwi head pose database compared to the ground truth.

Figure 13 shows the result of pose estimation in terms of yaw, depth, and roll for 10,000 frames from the Biwi head pose database compared to the ground truth.

A summary of the key evaluation results and method features of the proposed algorithm compared to two previous works is shown in Table 1. Three criteria were considered to compare the systems according to Pauly [40]:

- Accuracy: The 3D head tracker should estimate the head pose with a high precision compared to a ground truth. Note that this ground truth was generated by applying a third-party commercial software on a public dataset, on which we performed our experiments. Thus, the term ground truth is used just to be consistent with the literature and be able to compare with the previous works. In theory, any pose estimator including the commercial software should have some inaccuracy.
- Robustness: The 3D head tracker should be able to perform well under poor lighting conditions, fast motion of head and partial occlusion.
- Usability in real scenarios: User-specific training, system calibration and manual intervention need to be kept to a minimum. The tracker should be non-invasive. This will make the tracker be widely accepted by the users. Ideally, the system should be calibration-free.

On the one hand, our system demonstrates better results than [2,3] in terms of average error and standard deviation. Both systems proposed by Fanelli et al. show slightly better performance than our system *only* in terms of standard deviation of roll. Moreover, our system is generic and the training phase is performed with a single RGB/depth frame. On the other hand, the systems of Fanelli et al. can work on a frame-by-frame basis, while our system can only work in tracking mode (i.e., the subject’s head motion in successive frames should be small). Notice that both systems of Fanelli et al. need a training phase supported by a commercial face tracker, while we propose a new face tracker in this work. As both systems of Fanelli et al. require a training phase based on positive and negative patches cropped from a database of 20 subjects, the generic aspect of their system is an issue. We also compared our system to other non-model-based approaches [8,10,13,41], and the results show the effectiveness of the proposed system. The only comparable system is the model-based work of [11], which shows very good precision too.

#### 3.2.3. Gaze Estimation

##### Adaptive Linear Regression (ALR)

The results of testing the algorithm on the stationary head session of IDIAP database with 13 subjects are summarized in Table 2 and Table 3 for left eye and right eye, respectively, while Table 4 and Table 5 show the same results for the moving head session.

##### K-Nearest Neighbor

We used 40 training eye images from IDIAP database and represented the eye appearances as 15D feature vectors. These feature vectors are given to a K-NN regressor. Similar to Funes Mora and Odobez [7], we chose K=5. The steps of K-NN regression is as follows:

1. Given a test image, find the *K* closest set of sample images forming a neighborhood based on K-NN.
2. Find a set of weights: Inverse the distances of the K images from test image and normalize them.
3. Use the same weights to interpolate the parameters to obtain the estimated parameters for the test gazing point.

The results of testing the algorithm on the stationary head session of the IDIAP database with 13 subjects are summarized in Table 6 and Table 7 for the left eye and the right eye respectively, while Table 8 and Table 9 show the same results for the moving head session.

A summary of the key evaluation results and method features of the proposed algorithm compared to two previous works are shown in Table 10, Table 11, Table 12 and Table 13. Three criteria were considered to compare the systems:

- Accuracy compared to the ground truth data
- Robustness to occlusions, bad lighting and fast motions
- Usability in real scenarios, i.e., user-specific training, system calibration and manual intervention need to be kept to a minimum

Our system demonstrates better results than Funes and Odobez in terms of average gaze estimation error. One possible reason for this can be the high precession of our pose estimation system, which performs similar to a commercial state-of-the-art pose estimator, while the pose estimator of Kenneth and Odobez shows slight deviation from our precise pose estimator, which results in an imprecise texture warping on the head model that in turn can affect the gaze estimation process.

## 4. Conclusions

This work addressed the problem of automatic facial pose and gaze estimation without subject cooperation or manual intervention using low quality depth data provided by the Microsoft Kinect. Previous works on pose estimation using the Kinect are based on supervised learning or require manual intervention. In this work, we proposed a 3D pose estimator based on low quality depth data. The proposed method is generic and fully automatic. The experimental results on the Biwi head pose database confirm the efficiency of our algorithm in handling large head pose variations and partial occlusion. Our results also confirm that model-based approaches outperform other approaches in terms of precision. We also evaluated the performance of our algorithm on the IDIAP database for 3D eye gaze estimation (i.e., point of regard) and we obtained promising results. Although the feature-based gaze estimators are the most accurate ones in the literature, they require high resolution images. As Kinect has a low resolution RGB camera, we designed two appearance-based gaze estimators (i.e., manifold based ALR and K-NN) that do not rely on the local eye features. Instead, our proposed systems depend on the entire eye image content. Our gaze estimators outperformed the other appearance-based gaze estimators from several aspects, thanks to the high precision of the head tracker. Moreover, the user can freely turn their head, while this is not the case for most of the appearance-based methods, where the user should use a chin rest. 

## Figures and Tables

**Table 1:** A summary of the key evaluation results and method features of the proposed algorithm, and the two previous works based on supervised learning Fanelli et al. [2,3]. Notice that we do not compare the results with those of Funes and Odobez  [7] for face pose estimation due to lack of details in yaw, roll and pitch. Legend: 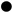, very good; 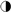, good;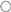, weak.

	Pose Estimation Error			Specifications		
	Pitch	Yaw	Roll	Accuracy	Robustness	Usability
Our Proposed Method	0.1 ± 6.7${}^{\circ }$	0.25 ± 8.7${}^{\circ }$	0.26 ± 9.3${}^{\circ }$	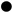	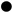	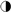
1st report [2]	8.5 ± 9.9${}^{\circ }$	8.9 ± 13.0${}^{\circ }$	7.9 ± 8.3${}^{\circ }$	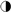	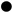	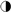
2nd report [3]	5.2 ± 7.7${}^{\circ }$	6.6 ± 12.6${}^{\circ }$	6.0 ± 7.1${}^{\circ }$	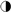	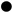	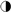
[13]	N/A	3.18 ± 5.3${}^{\circ }$	N/A	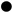	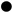	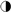
[10]	5.10${}^{\circ }$	6.29${}^{\circ }$	11.29${}^{\circ }$	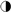	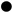	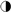
[8]	4.32 ± 2.65${}^{\circ }$	5.13 ± 3.33${}^{\circ }$	5.24 ± 3.33${}^{\circ }$	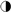	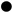	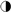
[12]	2.5 ± 7.4${}^{\circ }$	3 ± 9.6${}^{\circ }$	3.8 ± 16${}^{\circ }$	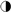	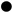	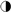
[41]	2.54${}^{\circ }$	2.57${}^{\circ }$	3.62${}^{\circ }$	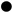	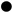	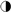
[11]	1.7${}^{\circ }$	2.5${}^{\circ }$	2.3${}^{\circ }$	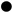	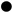	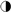

**Table 2:** Left eye, Adaptive Linear Regression, stationary head.

Subject	Mean	Median	Min	Max	Std. Dev.
1.0000	6.5668	5.5213	0.0656	28.5774	4.5051
2.0000	6.5204	5.2114	0.1009	30.3382	4.9035
3.0000	5.2308	4.1809	0.0343	32.3251	4.1530
4.0000	7.6606	6.2322	0.0343	33.4197	5.6697
5.0000	8.8390	7.4393	0.1690	36.2075	5.8689
6.0000	8.3817	6.9423	0.0839	32.3026	5.7648
8.0000	7.6642	5.6913	0.0593	34.5903	6.0913
9.0000	8.5617	7.7270	0.0343	28.0875	4.8624
10.0000	7.0422	6.0081	0.0593	33.9498	4.8673
11.0000	6.0904	4.9469	0.0280	30.9162	4.4417
14.0000	5.9717	5.4238	0.0885	26.0122	3.5437
15.0000	14.8658	4.9548	0.1009	179.5907	38.0497
16.0000	4.7865	3.7544	0.0740	30.7344	4.0378

**Table 3:** Right eye, Adaptive Linear Regression, stationary head.

Subject	Mean	Median	Min	Max	Std. Dev.
1.0000	6.5626	5.1144	0.0713	32.5561	5.2122
2.0000	6.4349	5.0625	0.0907	31.7140	5.0184
3.0000	6.2339	4.2259	0.1047	33.9836	5.4509
4.0000	8.4404	6.6909	0.0907	32.5826	6.2366
5.0000	8.9205	7.4186	0.2839	35.7794	6.0221
6.0000	7.7985	5.9969	0.0442	35.7014	5.9868
8.0000	6.7669	5.4983	0.0626	37.1941	4.9395
9.0000	8.9776	8.1380	0.1136	30.3615	5.2164
10.0000	6.0015	4.8519	0.0396	32.2359	4.6771
11.0000	5.5846	3.9305	0.0442	30.4535	5.0931
14.0000	6.1953	5.6908	0.0928	26.0149	3.4915
15.0000	5.8307	4.8741	0.0343	28.7052	4.2100
16.0000	5.8875	4.4554	0.0396	32.4213	4.8225

**Table 4:** Left eye, Adaptive Linear Regression, moving head.

Subject	Mean	Median	Min	Max	Std. Dev.
1.0000	14.9208	12.3744	0.1187	63.5577	10.5413
2.0000	10.0484	8.6127	0.0685	41.7107	6.5863
3.0000	10.6290	8.5283	0.0198	52.6725	8.1252
4.0000	13.6127	11.2650	0.1736	49.9972	9.6486
5.0000	8.6551	7.2148	0.0560	50.2660	6.0730
6.0000	9.1024	8.0921	0.0343	33.3412	5.6728
8.0000	13.6427	10.7772	0.1267	53.1464	9.8346
9.0000	9.6416	7.7782	0.0766	46.3817	7.1335
10.0000	9.2250	7.3112	0.0593	43.3608	7.0226
11.0000	7.8273	5.8934	0.1065	52.2461	6.7665
14.0000	5.1753	4.3553	0.1028	25.9897	3.7616
15.0000	7.7078	6.4144	0.1187	29.6265	5.2408
16.0000	6.8988	4.9801	0.1791	38.4438	5.7533

**Table 5:** Right eye, Adaptive Linear Regression, moving head.

Subject	Mean	Median	Min	Max	Std. Dev.
1.0000	14.3154	11.9387	0.1101	58.8258	10.0281
2.0000	9.5803	8.1392	0.1494	46.8254	6.4423
3.0000	9.9308	7.8859	0.1342	48.7736	7.5894
4.0000	12.4269	9.9770	0.0656	50.1240	9.2286
5.0000	9.8638	8.4771	0.2647	48.7462	6.3292
6.0000	6.6662	5.5380	0.0560	33.5227	4.7564
8.0000	14.3784	10.4478	0.0280	53.7265	11.5794
9.0000	9.4942	7.5957	0.0626	44.8285	6.9502
10.0000	9.0722	6.6025	0.0560	46.5259	7.5245
11.0000	7.5377	5.8438	0.0685	43.7269	6.1082
14.0000	5.1321	4.1259	0.1028	24.8975	3.8079
15.0000	7.5045	6.5732	0.0485	32.9121	4.5594
16.0000	7.4444	5.1954	0	36.1109	6.5095

**Table 6:** Left eye, KNN, stationary.

Subject	Mean	Median	Min	Max	Std. Dev.
1.0000	6.2637	5.6606	0.0485	29.3913	3.8814
2.0000	6.7424	5.6652	0.0343	29.7500	4.5975
3.0000	5.4487	4.4840	0.0656	32.3594	4.0323
4.0000	8.0647	7.1761	0.0343	29.9259	4.9577
5.0000	8.7174	7.6801	0.2812	30.9190	5.1745
6.0000	7.9754	6.7632	0.0928	33.2063	5.4553
8.0000	6.8706	6.2672	0.0560	34.9599	4.1641
9.0000	8.3298	7.8083	0.1845	23.8339	4.1954
10.0000	6.7440	5.7991	0.1084	31.7058	4.4667
11.0000	5.9657	4.8900	0.1065	29.5153	4.2912
14.0000	5.7215	5.0924	0.1297	24.6203	3.4290
15.0000	32.1286	5.1132	0.0280	179.4339	62.0019
16.0000	5.7970	5.0794	0.0523	30.8534	3.8672

**Table 7:** Right eye, KNN, stationary.

Subject	Mean	Median	Min	Max	Std. Dev.
1.0000	6.1009	5.3165	0.0816	27.7014	3.9828
2.0000	6.6069	5.7964	0.0442	31.1315	4.2246
3.0000	4.9409	4.0003	0.0396	31.6954	3.8844
4.0000	7.8655	6.9778	0.0198	30.8593	4.7412
5.0000	8.0041	7.3985	0.2037	30.7498	4.5424
6.0000	7.3753	6.1297	0.0791	34.9256	5.0427
8.0000	6.6605	6.1925	0.1570	34.7294	3.9806
9.0000	8.4486	7.7163	0.0713	26.2216	4.6055
10.0000	5.9578	4.7960	0.2533	31.6315	4.4807
11.0000	5.5172	4.3471	0.0560	30.7713	4.1658
14.0000	6.0244	5.3324	0.0969	25.0551	3.7456
15.0000	5.0204	4.1662	0.0685	28.7128	3.8207
16.0000	5.8353	5.1276	0.0280	28.7520	3.7264

**Table 8:** Left eye, KNN, moving.

Subject	Mean	Median	Min	Max	Std. Dev.
1.0000	12.6972	10.6383	0.0791	62.4921	9.0053
2.0000	9.2681	7.9303	0.0949	46.0430	6.3844
3.0000	10.0220	8.4790	0.2037	50.2988	7.1276
4.0000	12.4382	10.7494	0.2698	47.8893	8.1049
5.0000	8.6143	7.1774	0.1824	124.3536	6.0929
6.0000	8.6792	7.6599	0.1938	35.7626	5.0370
8.0000	10.2691	8.2749	0.1643	40.4763	7.3319
9.0000	9.4805	7.8364	0.0560	46.4132	6.6735
10.0000	8.7826	7.1364	0.1136	56.2227	6.7277
11.0000	7.0845	5.9344	0.0656	57.5333	5.1368
14.0000	5.6254	4.7095	0.0280	26.2963	3.8907
15.0000	7.8371	6.6540	0.1494	31.0181	4.9755
16.0000	6.5743	5.6239	0.1009	34.7607	4.3908

**Table 9:** Right eye, KNN, moving.

Subject	Mean	Median	Min	Max	Std. Dev.
1.0000	12.3237	10.4269	0.1887	45.3387	7.9340
2.0000	8.9763	7.7712	0.2056	38.0011	5.8696
3.0000	9.0502	7.9296	0.0791	40.7372	6.0890
4.0000	11.2250	9.1458	0.1413	123.2889	8.1434
5.0000	9.3295	8.7252	0.2149	45.1831	5.4824
6.0000	7.0146	6.4300	0.1342	33.4972	4.0739
8.0000	12.1559	8.8643	0.0713	77.7315	12.3338
9.0000	9.4243	7.5941	0.2194	45.4437	6.9684
10.0000	8.7082	6.8501	0.1119	48.6272	7.0996
11.0000	7.2499	6.1685	0.0485	48.6069	5.3183
14.0000	5.3395	4.4847	0.1558	24.1723	3.5878
15.0000	7.5671	6.8155	0.1327	33.6782	4.4902
16.0000	6.8581	5.0058	0.1327	40.4460	5.8367

**Table 10:** A summary of the key evaluation results and method features of the proposed algorithm, and the previous work when Adaptive Linear Regression (ALR) is used and the subjects keep the head stationary: 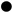, very good; 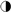, good;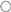, weak.

	Gaze Estimation Error		Specifications		
	Left Eye	Right Eye	Accuracy	Robustness	Usability
Our Proposed Method	7.55${}^{\circ }$	6.89${}^{\circ }$	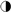	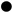	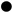
Funes and Odobez Method	9.73${}^{\circ }$	10.5${}^{\circ }$	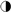	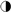	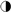

**Table 11:** A summary of the key evaluation results and method features of the proposed algorithm, and the previous work when K-NN is used and the subject keeps the head stationary: 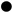, very good; 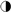, good;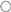, weak.

	Gaze Estimation Error		Specifications		
	Left Eye	Right Eye	Accuracy	Robustness	Usability
Our Proposed Method	8.83${}^{\circ }$	6.49${}^{\circ }$	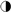	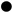	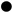
Funes and Odobez Method	10.23${}^{\circ }$	9.56${}^{\circ }$	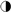	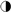	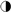

**Table 12:** A summary of the key evaluation results and method features of the proposed algorithm, and the previous work when Adaptive Linear Regression (ALR) is used and the subjects have free head motion: ⚫, very good; 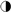, good;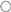, weak.

	Gaze Estimation Error		Specifications		
	Left Eye	Right Eye	Accuracy	Robustness	Usability
Our Proposed Method	9.78${}^{\circ }$	9.49${}^{\circ }$	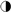	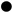	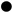
Funes and Odobez Method	15.57${}^{\circ }$	14.2${}^{\circ }$	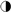	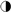	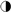

**Table 13:** A summary of the key evaluation results and method features of the proposed algorithm, and the previous work when K-NN is used and the subjects have free head motion: 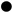, very good; 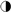, good;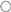, weak.

	Gaze Estimation Error		Specifications		
	Left Eye	Right Eye	Accuracy	Robustness	Usability
Our Proposed Method	9.03${}^{\circ }$	8.86${}^{\circ }$	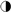	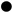	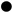
Funes and Odobez Method	17.97${}^{\circ }$	14.63${}^{\circ }$	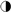	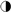	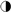

## References

[1] Murphy-Chutorian E., Trivedi M.M. (2009). Head pose estimation in computer vision: A survey. IEEE Trans. Pattern Anal. Mach. Intell..

[2] Fanelli G., Weise T., Gall J., Van Gool L. (2011). Real time head pose estimation from consumer depth cameras. Pattern Recognition.

[3] Fanelli G., Dantone M., Gall J., Fossati A., Van Gool L. (2013). Random Forests for Real Time 3D Face Analysis. Int. J. Comput. Vis..

[4] Breitenstein M.D., Kuettel D., Weise T., Van Gool L., Pfister H. Real-time face pose estimation from single range images. Proceedings of the 2008 IEEE Conference on Computer Vision and Pattern Recognition.

[5] Fanelli G., Gall J., Van Gool L. Real time head pose estimation with random regression forests. Proceedings of the CVPR 2011.

[6] Seemann E., Nickel K., Stiefelhagen R. Head pose estimation using stereo vision for human-robot interaction. Proceedings of the Sixth IEEE International Conference on Automatic Face and Gesture Recognition.

[7] Funes Mora K.A., Odobez J. Gaze estimation from multimodal Kinect data. Proceedings of the 2012 IEEE Computer Society Conference on Computer Vision and Pattern Recognition Workshops.

[8] Rekik A., Ben-Hamadou A., Mahdi W. 3D Face Pose Tracking using Low Quality Depth Cameras. Proceedings of the International Conference on Computer Vision Theory and Applications—VISAPP 2013.

[9] Cai Q., Gallup D., Zhang C., Zhang Z. (2010). 3D Deformable Face Tracking with a Commodity Depth Camera. Proceedings of the 11th European Conference on Computer Vision Conference on Computer Vision: Part III, ECCV’10.

[10] Baltrusaitis T., Robinson P., Morency L.P. 3D Constrained Local Model for rigid and non-rigid facial tracking. Proceedings of the 2012 IEEE Conference on Computer Vision and Pattern Recognition.

[11] Yu Y., Mora K.A.F., Odobez J.M. Robust and Accurate 3D Head Pose Estimation through 3DMM and Online Head Model Reconstruction. Proceedings of the 2017 12th IEEE International Conference on Automatic Face Gesture Recognition (FG 2017).

[12] Papazov C., Marks T.K., Jones M. Real-time 3D head pose and facial landmark estimation from depth images using triangular surface patch features. Proceedings of the 2015 IEEE Conference on Computer Vision and Pattern Recognition (CVPR).

[13] Tulyakov S., Vieriu R.L., Semeniuta S., Sebe N. Robust Real-Time Extreme Head Pose Estimation. Proceedings of the 2014 22nd International Conference on Pattern Recognition.

[14] Hansen D., Ji Q. (2010). In the Eye of the Beholder: A Survey of Models for Eyes and Gaze. IEEE Trans. Pattern Anal. Mach. Intell..

[15] Villanueva A., Cabeza R., Porta S. (2006). Eye tracking: Pupil orientation geometrical modeling. Image Vis. Comput..

[16] Wang J.G., Sung E., Venkateswarlu R. (2005). Estimating the eye gaze from one eye. Comput. Vis. Image Underst..

[17] Villanueva A., Cabeza R., Porta S. (2007). Gaze Tracking System Model Based on Physical Parameters. IJPRAI Int. J. Pattern Recognit. Artif. Intell..

[18] White K.P., Hutchinson T.E., Carley J.M. (1993). Spatially dynamic calibration of an eye-tracking system. IEEE Trans. Syst. Man Cybern..

[19] Guestrin E.D., Eizenman M. (2006). General theory of remote gaze estimation using the pupil center and corneal reflections. IEEE Trans. Biomed. Eng..

[20] Morimoto C.H., Amir A., Flickner M. Detecting eye position and gaze from a single camera and 2 light sources. Proceedings of the Object Recognition Supported by User Interaction for Service Robots.

[21] Blignaut P. (2013). Mapping the pupil-glint vector to gaze coordinates in a simple video-based eye tracker. J. Eye Mov. Res..

[22] Cherif Z.R., Nait-Ali A., Motsch J., Krebs M. (2002). An adaptive calibration of an infrared light device used for gaze tracking. Proceedings of the 19th IEEE Instrumentation and Measurement Technology Conference, IMTC/2002.

[23] Brolly X.L., Mulligan J.B. (2004). Implicit calibration of a remote gaze tracker. Proceedings of the 2004 Conference on Computer Vision and Pattern Recognition Workshop, CVPRW’04.

[24] Cerrolaza J.J., Villanueva A., Cabeza R. (2008). Taxonomic study of polynomial regressions applied to the calibration of video-oculographic systems. Proceedings of the 2008 Symposium on Eye Tracking Research & Applications.

[25] Zhu Z., Ji Q. (2004). Eye and gaze tracking for interactive graphic display. Mach. Vis. Appl..

[26] Chi J., Zhang C., Yan Y., Liu Y., Zhang H. (2009). Eye gaze calculation based on nonlinear polynomial and generalized regression neural network. Proceedings of the Fifth International Conference on Natural Computation, ICNC’09.

[27] Ma C., Choi K.A., Choi B.D., Ko S.J. (2015). Robust remote gaze estimation method based on multiple geometric transforms. Opt. Eng..

[28] Pomerleau D., Baluja S. Non-intrusive gaze tracking using artificial neural networks. Proceedings of the AAAI Fall Symposium on Machine Learning in Computer Vision.

[29] Tan K.H., Kriegman D.J., Ahuja N. (2002). Appearance-based eye gaze estimation. Proceedings of the Sixth IEEE Workshop on Applications of Computer Vision (WACV 2002).

[30] Lu F., Sugano Y., Okabe T., Sato Y. Inferring human gaze from appearance via adaptive linear regression. Proceedings of the 2011 IEEE International Conference on Computer Vision (ICCV).

[31] Williams O., Blake A., Cipolla R. (2006). Sparse and Semi-supervised Visual Mapping with the S^ 3GP. Proceedings of the 2006 IEEE Computer Society Conference on Computer Vision and Pattern Recognition.

[32] Noris B., Keller J.B., Billard A. (2011). A wearable gaze tracking system for children in unconstrained environments. Comput. Vis. Image Underst..

[33] Choi D.H., Jang I.H., Kim M.H., Kim N.C. (2007). Color image enhancement based on single-scale retinex with a JND-based nonlinear filter. Proceedings of the IEEE International Symposium on Circuits and Systems, ISCAS 2007.

[34] Funes-Mora K.A., Odobez J.M. (2016). Gaze estimation in the 3d space using rgb-d sensors. Int. J. Comput. Vis..

[35] Viola P., Jones M. Rapid object detection using a boosted cascade of simple features. Proceedings of the 2001 IEEE Computer Society Conference on Computer Vision and Pattern Recognition, CVPR 2001.

[36] Bouaziz S., Pauly M. (2013). Dynamic 2d/3d Registration for the Kinect.

[37] LaValle S.M. (2006). Planning Algorithms.

[38] Bouaziz S., Tagliasacchi A., Pauly M. Sparse iterative closest point. Proceedings of the Eleventh Eurographics/ACMSIGGRAPH Symposium on Geometry Processing.

[39] Funes Mora K.A., Monay F., Odobez J.M. (2014). EYEDIAP Database: Data Description and Gaze Tracking Evaluation Benchmarks.

[40] Pauly M. Realtime Performance-Based Facial Avatars for Immersive Gameplay. Proceedings of the Motion on Games.

[41] Martin M., van de Camp F., Stiefelhagen R. Real Time Head Model Creation and Head Pose Estimation on Consumer Depth Cameras. Proceedings of the 2014 2nd International Conference on 3D Vision.

